# SPOUSAL ASSOCIATIONS BETWEEN GRANDPARENT CAREGIVING AND WELL-BEING: FINDINGS FROM THE HEALTH AND RETIREMENT STUDY

**DOI:** 10.1093/geroni/igac059.2108

**Published:** 2022-12-20

**Authors:** Athena Chung Yin Chan, Rodlescia Sneed

**Affiliations:** University of Minnesota--Twin Cities, Minneapolis, Minnesota, United States; Wayne State University, Detroit, Michigan, United States

## Abstract

Numerous studies document the impact of grandparent caregiving on the health and well-being of grandparents; however, there has been little dyadic research on how the caregiving and health-related outcomes of one grandparent influence partner couples The purpose of this study was to determine the interdependence of grandparents’ intensity of caregiving and well-being (i.e., depressive symptoms and self-rated health) over time. Participants were 7,133 dyads of American grandparents aged ≥ 50 who participated in the Health and Retirement Study, a population-based study of community-dwelling adults, in 2010 and 2012. Data on hours of grandparent caregiving in the past two years, depressive symptoms, and self-rated health were obtained via self-report. Two longitudinal, dyadic path analyses were conducted using the Actor-Partner Interdependence Model. Within individuals (actor effects), greater depressive symptoms and better self-rated health at baseline, predicted greater depressive symptoms and better self-rated health two years later. Between spouses (partner effects), an individuals’ greater depressive symptoms predicted the spouses’ greater depressive symptoms. However, grandfathers’ better self-rated health predicted subsequent better grandmothers’ self-rated health, but not vice versa. Further, greater depressive symptoms among grandmothers predicted lower subsequent caregiving intensity among both grandmothers and grandfathers. Additionally, better self-rated health among grandfathers predicted better self-rated health and lower subsequent self and spousal grandparenting caregiving intensity. Our findings demonstrate that depressive symptoms, self-rated health, and caregiving intensity are interrelated among grandparent couples. Interventions for improving well-being and caregiving outcomes that focus on couples may be more effective than those that focus on individuals.

